# Draft Whole-Genome Sequences of Three Isolates of a Novel Strain of a *Campylobacter* sp. Isolated from New Zealand Birds and Water

**DOI:** 10.1128/MRA.00258-19

**Published:** 2019-05-02

**Authors:** David A. Wilkinson, Anne C. Midwinter, Errol Kwan, Samuel J. Bloomfield, Nigel P. French, Patrick J. Biggs

**Affiliations:** a School of Veterinary Science, Massey University, Palmerston North, New Zealand; b New Zealand Food Safety Science and Research Centre, Massey University, Palmerston North, New Zealand; c Quadram Institute, Norwich, United Kingdom; d School of Fundamental Sciences, Massey University, Palmerston North, New Zealand; University of Southern California

## Abstract

*Campylobacter* spp. are frequently found associated with the avian intestinal tract. Most are commensals, but some can cause human campylobacteriosis. Here, we report the draft genome sequences of three strains of a novel *Campylobacter* sp. isolated from urban birds and a rural river in New Zealand.

## Abstract (precis)

*Campylobacter* spp. are frequently found associated with the avian intestinal tract. Most are commensals, but some can cause human campylobacteriosis.

## ANNOUNCEMENT

*Campylobacter* species are frequent commensals of the avian gastrointestinal tract, and C. jejuni isolated from birds is associated with zoonotic campylobacteriosis in humans (1, 2). Additionally, wild birds are a rich source of novel *Campylobacter* species (3, 4). Swabs of deposited feces from starlings and mallard ducks in Palmerston North, Manawatu, New Zealand, as well as a 0.45-μm mixed cellulose ester filter (Millipore, Germany) of 100 ml of water from the Pareora River in Canterbury, New Zealand, were enriched in Bolton broth (LabM, Hampshire, UK) in a microaerobic atmosphere at 42°C and subcultured onto modified charcoal-cefoperazone-deoxycholate agar (mCCDA) (Fort Richard Laboratories, Auckland, New Zealand). Three strains of nonstandard colonial morphology (B423b from a mallard duck, B1491 from a starling, and W677a from the Pareora River) had crude DNA extracted by boiling and 16S rRNA PCR performed using the methodology of Linton and coauthors (5). The products were Sanger sequenced at the Massey Genome Service (Massey University, Palmerston North, New Zealand). Genomic DNA was extracted from a single colony using the QIAamp DNA minikit (Qiagen, Germany) for W677a and the Wizard genomic DNA kit (Promega, WI) for B423b and B1491. DNA was sequenced at New Zealand Genomics Ltd. (Massey University) using a MiSeq instrument (Illumina, Inc.) with paired read lengths of 250 base pairs after library preparation using the Nextera XT library kit (Illumina). Sequence data were trimmed, assembled, and annotated using the “reads to report” Nullarbor pipeline (https://github.com/tseemann/nullarbor), which uses Trimmomatic with default settings (6) and SPAdes v.3.9.0 in careful mode (7). Annotation statistics were extracted from the NCBI Prokaryotic Genome Annotation Pipeline (PGAP) after being uploaded to the NCBI server and are described in Table 1.

**TABLE 1:** Campylobacter sp. strain data

Strain	Length (bp)	No. of contigs	*N*_50_ (bp)	No. of reads	Coverage (×)	No. of CDS^*a*^	GC content (%)	Assembly accession no.	GenBank accession no.	SRA accession no.
W677a	1,638,711	37	87,075	859,904	81	1,647	27.48	GCA_004323835	QPGP00000000	SRR8367113
B1491	1,647,568	28	77,232	2,092,424	308	1,666	27.44	GCA_004323825	QPGQ00000000	SRR8367114
B423b	1,571,819	59	119,547	776,820	115	1,614	27.45	GCA_004323845	QPGR00000000	SRR8367115

a CDS, coding DNA sequences.

Similarity to known *Campylobacter* spp. was measured by BLAST analysis of the 16S rRNA gene, and genomic average nucleotide identity (ANI) was calculated using the Kosta laboratory’s ANI calculator (http://enve-omics.ce.gatech.edu/ani/). The three presented isolates showed 99% ANI and 100% 16S pairwise identity compared to each other, suggesting a monophyletic lineage. Both BLAST and ANI methods identified the most closely related species as being members of C. jejuni, showing 98.15% 16S pairwise identity to C. jejuni NCTC13268 and ∼79% ANI to C. jejuni (GenBank accession number GCA_000011865), suggesting that the isolates are likely members of a previously undescribed taxonomic group.

The isolates had a genome with an estimated mean size of 1.62 Mb ± 0.04 Mb standard deviation and 1,642 (±26) predicted coding sequences. The average GC content was 27.46% (±0.02%). This is lower than that observed in other *Campylobacter* spp., which typically falls between 30 and 46% (8), with the previously described lowest being 27.9% for C. hepaticus (9). Single copies of 5S, 16S, and 23S rRNA genes were identified in each genome. ABRicate (https://github.com/tseemann/abricate) was used to query the Virulence Factors Database (10) using default settings. Neither the *cdtABC* operon responsible for cytolethal distending toxin production nor the pVir plasmid associated with described pathogenicity in other *Campylobacter* species was found, suggesting that these isolates potentially belong to an avirulent commensal taxonomic group.

### Data availability.

The genome assemblies have been deposited in GenBank, and the accession numbers are detailed in Table 1.
